# Assessment of knowledge and self-efficacy among health professionals and clinical scientists following the Cameroon HIV/AIDS Research Forum (CAM-HERO 2022) research methodology and bioethics training

**DOI:** 10.11604/pamj.2024.47.91.41870

**Published:** 2024-02-27

**Authors:** Peter Vanes Ebasone, Appolinaire Tiam, Patrice Tchendjou, Merveille Foaleng, Eveline Mboh Khan, Rogers Ajeh, Boris Tchounga, Emile Nforbih Shu, Gabriel Mabou, Johney Melpsa, Pius Tih Muffih, Andre Pascal Kengne, Anne Cecile Zoung-Kany Bisseck, Anastase Dzudie, Friedrich Thienemann

**Affiliations:** 1 Clinical Research Education, Networking and Consultancy (CRENC), Yaoundé, Cameroon,; 2 Department of Medicine, Faculty of Health Science, University of Cape Town, Cape Town, South Africa,; 3 Elizabeth Glaser Pediatric AIDS Foundation, Washington, DC, USA,; 4 Elizabeth Glaser Pediatric AIDS Foundation (EGPAF), Yaoundé, Cameroon,; 5 Cameroon Baptist Convention Health Services (CBCHS), Bamenda, Cameroon,; 6 National AIDS Control Committee, Ministry of Public Health, Yaoundé, Cameroon,; 7 Non-Communicable Diseases Research Unit, South African Medical Research Council, Cape Town, South Africa,; 8 Division of Health Operational Research, Ministry of Public Health, Yaoundé, Cameroon,; 9 Faculty of Medicine and Biomedical Sciences, University of Yaoundé I, Yaoundé, Cameroon,; 10 Department of Internal Medicine and Subspecialties, Douala General Hospital, Douala, Cameroon,; 11 Lown Scholars Program, Department of Global Health and Population, Harvard T.H. Chan School of Public Health, Boston, USA,; 12 General Medicine & Global Health, Department of Medicine and Cape Heart Institute, Faculty of Health Science, University of Cape Town, Cape Town, South Africa,; 13 Department of Internal Medicine, University Hospital Zurich, University of Zurich, Zurich, Switzerland

**Keywords:** Cameroon, Africa, knowledge assessment, self-efficacy, research methodology, health professionals

## Abstract

To inform public health policymakers that the generation of local evidence-based knowledge is key. Research capacity in low- and middle-income countries (LMIC) to generate medical knowledge is often weak and insufficiently resourced and efforts to tackle these challenges are not standardized. Continuous research training can equip researchers with the required knowledge and research skills, but its effectiveness largely depends on the quality and pertinence of the training methods used. We aim to assess the effectiveness of the Cameroon HIV/AIDS Research Forum (CAM-HERO) 2022 Research Methodology and Bioethics Training with the objective to describe the knowledge gained and the self-efficacy of health professionals and clinical scientists. A survey was conducted during the one-day training among health professionals and clinical scientists. Participants took an online self-administered questionnaire before and after the training related to the topics taught. The questionnaire consisted of two parts: 1) 18 Multiple Choice Questions (MCQs) to assess knowledge and 2) Nine items to evaluate self-efficacy using a five-point Likert scale. Mean scores were calculated, analysed, and compared using paired t-test for the pre- and post-test results. A total of 30 participants (57% women) completed the socio-demographic form. The median age (IQR) of participants was 33.5 (13.3) years. We registered 38 respondents for the pre-test and 33 respondents for the post-test. There was a rise in knowledge mean score from 13.0 to 14.8 (p=0.001) and an improvement in the perception of self-efficacy with a mean score increase from 2.9 to 3.7 (p < 0.001). Knowledge and perception of self-efficacy on research methodology improved among participants after the training. These results suggest that the CAM-HERO 2022 training had an immediate positive impact on skills and self-efficacy. Hence, we recommend the implementation of this training on a larger scale, periodically, and with long-term follow-up to evaluate its impact.

## Introduction

Research plays a crucial role in understanding, describing, predicting, and explaining health phenomena, making it an indispensable tool for health professionals in both academic and clinical settings [1]. Having proficiency in research methodology can lead to the discovery of innovative technologies and evidence-based medicine that can significantly impact the field of science and improve patient care. The research community is broad and encompasses multiple disciplines and educational backgrounds. Researchers must possess sound knowledge of both the theoretical and practical aspects of research methods to practice good research. Some experts have expressed concerns over the lack of basic research skills among young researchers [2]. Keeping up-to-date with constantly evolving knowledge is important to replace outdated practices [3]. Unfortunately, for many reasons including limited training and lack of leadership, the research capacity in low- and middle-income countries (LMIC) is weak [4,5]. Consequently, there is a shortage of scientifically sound and updated research in sub-Saharan Africa [4].

Few studies have investigated the impact of training interventions on research methodology and their significance in building and strengthening research skills for both early-career and experienced health professionals in Cameroon. The Cameroon HIV Research Forum (CAM-HERO) is a periodic meeting of local and international scientists and clinicians, policymakers, and regulatory authorities; a platform of a range of researchers, independent research organizations, and research regulation authorities which aims at improving the relevance, performance, and impact of research in Cameroon with a focus on HIV/AIDS. One of its goals is to enhance research capacity among local researchers. Based on observations of the needs from the previous two conferences, the third CAM-HERO conference and first training on research methodology and bioethics took place in December 2022 in Kribi, Cameroon with the aim to equip participants with the theoretical concepts and best practices on the development of a research question, hypothesis generation, ethical and regulatory concepts, epidemiological study designs and basic bio-statistical methods in health research [5]. In this manuscript, we report the evaluation of the effectiveness of this research training on research methodology knowledge and self-efficacy of participants.

## Workshop report

**Location:** CAM-HERO arranged and supported the workshop, which took place on the 01^st^ of December 2022, at Hotel Le Lagon Resort, Kribi, Cameroon.

**Aim and objectives:** the purpose of the workshop was to build research capacity of local and international scientists and clinicians, policymakers, and regulatory authorities to 1) disseminate HIV research findings and HIV policy; 2) foster operational research collaboration; 3) initiate a guideline for promoting HIV/AIDS research in Cameroon. The specific objectives included: 1) assess learner´s problems and introduce learners to faculty members; 2) differentiate between a research topic, a goal, and a research question and understand the characteristics of a good research question; 3) understand research designs and their application in HIV research; 4) understand the research design and methods in clinical trials; 5) understand the basis of data management, statistical analysis and scientific writing.

### Facilitators

Workshop facilitators brought a wealth of multidisciplinary experience. Facilitators included a Cameroonian professor of cardiology and epidemiology from the Clinical Research Education Networking and Consultancy, in Yaoundé Cameroon, the Faculty of Medicine and Pharmaceutical Sciences of the University of Yaoundé I and the Department of Internal Medicine and Subspecialties of the Douala General Hospital in Cameroon. A South African professor of internal medicine and infectious diseases from the Department of Medicine, Faculty of Health Science at the University of Cape Town in South Africa and the Department of Internal Medicine, University Hospital Zurich of the University of Zurich in Switzerland. A Cameroonian doctor and epidemiologist from the Elizabeth Glaser Pediatric AIDS Foundation, Washington, United States of America and another Cameroonian Doctor from the Elizabeth Glaser Pediatric AIDS Foundation, Yaoundé, Cameroon. A Cameroonian doctor and epidemiologist from the National AIDS Control Committee at the Ministry of Public Health in Yaoundé, Cameroon. A South African professor of internal medicine and epidemiology from the Department of Medicine, Faculty of Health Science, University of Cape Town, in South Africa and the Non-Communicable Diseases Research Unit, South African Medical Research Council in Cape Town, South Africa. A Cameroonian professor of epidemiology from the Cameroon Baptist Convention Health Services, Bamenda, Cameroon and a Cameroonian professor of dermatology from the Division of Health Operational Research, Ministry of Public Health, Yaoundé, Cameroon and the Faculty of Medicine and Biomedical Sciences, University of Yaoundé I, Yaoundé, Cameroon. Participants from various universities and organizations shared their experiences through formal presentations and discussions.

**Selection of participants:** prior to the training, a call for applications was opened on the CAM-HERO website to select health science students, researchers and professionals with an interest or experience in public health projects and clinical research. A scientific committee reviewed the applications and eligible participants were selected based on developed and adopted minimum criteria. Out of 91 applications received, 35 (38.5%) were selected based on their research profiles and achievements.

**Online Pre-workshop test:** a 27-questions multiple-choice test with correct/incorrect answers and a five-point Likert scale was designed to assess knowledge (Q1-Q18) and self-efficacy (item 1 to 9) (the list of questions is shown in Annex 1). Self-efficacy was defined as a participant´s belief in his/her ability to possess and execute the necessary skills to produce specific performance attainments in research methods. The questions were applicable to the training topics and approved by experienced trainers. A separate questionnaire was designed and used to gather participants' sociodemographic information.

### Program and training methodology

The training was a pivotal component of the CAM-HERO 2022 HIV conference, held on 1^st^ December at Hotel Le Lagon Resort, Kribi. To foster a more engaging and interactive learning environment, both the trainers and trainees were situated in a U-shaped seating arrangement. This allowed for improved visibility, enhanced interaction, and overall, a better learning experience. Before the commencement of the training, a QR code was projected on the screen. These enabled participants to access and submit their responses to the pre-test online, thereby providing a baseline measure of the learners' knowledge and understanding of the topics to be discussed. The training utilized an instructor-led approach, but was not unidirectional. Trainers conducted their sessions while encouraging active learner participation. This instructional method incorporated multiple training strategies. Each instructor had a 45-minute PowerPoint presentation that offered a structured overview of the topic. This was then followed by interactive sessions where learners could ask questions and engage in discussions with the trainers. The curriculum was diverse, covering aspects such as formulating research questions, understanding various research designs, ethical concepts, clinical trials, descriptive statistics, and scientific writing. Teaching strategies included theoretical presentations complemented by discussions of practical aspects to reinforce understanding. After the training, a post-test, mirroring the pre-test, was administered. This allowed us to gauge the participants' knowledge acquisition and assess the effectiveness of our training methodology.

**Statistical analysis:** data analysis used SPSS version 25.0. Percentages and frequencies were calculated for the sociodemographic characteristics of participants. The knowledge assessment responses (pre and post) were coded “1” for correct responses and “0” for incorrect responses. Means and standard deviations for pre- and post-knowledge assessment and self-efficacy evaluation were calculated overall, per section and per question. We equally evaluated the percentage change in mean across the training for each question in the following order: 0-25% = Zero, 26-50% = Average, 51-75% = Good, 76-100% = Excellent. To compare means between pre and post-test, a paired Student´s t-test was used. P-values less than 0.05 were considered statistically significant.

### Evaluation

**Sociodemographic characteristics:** Table 1 describes the sociodemographic characteristics of respondents. Thirty-seven percent (37%) of the respondents were medical doctors, while others were nurses and scientists; 17 (57%) were women. The age of participants ranged from 23 to 55 years with a median (IQR) age of 33.5 (13.3) years. Fourteen (47%) of them reported to have already completed a master´s level, and 25 (83%) had already been involved in a research study before. Thirteen (43%) had never attended a research training, and 18 (60%) had never presented at a conference. Twenty-one (70%) respondents had already written an abstract or authored/co-authored a research paper.

**Table 1:** sociodemographic characteristics of respondents

Variable	Results 1 (N=30)
**Gender**	
Male	13(43.3)
Female	17(56.7)
**Highest level of education**	
Higher Diploma in State Registered Nurse	3(10)
Master of Science	14(46.7)
General practitioner (MD)	8(26.7)
PhD	2(6.7)
Specialist physician	3(10)
**Have you ever been involved in a research study?**	
No	5(16.7)
Yes	25(83.3)
**Have you attended any research training apart from CAM-HERO 2022?**	
No	13(43.3)
Yes	17(56.7)
**Have you ever written an abstract?**	
No	9(30)
Yes	21(70)
**Have you presented in a research conference apart from CAM-HERO 2022?**	
No	18(60)
Yes	12(40)
**Have you authored or co-authored an original research paper?**	
No	9(30)
Yes	21(70)

[1] The values represent the frequency and percentage for the variables n (%)

**Knowledge assessment:** thirty-eight (100%) participants completed the pre-test, and 33 (86.8%) participants completed the post-test. Overall, the mean (SD) pre-test and post-test scores were 13.0 (2.5) and 14.8 (1.9), respectively. The mean difference between the two tests was 1.8 (p = 0.001). Table 2 describes the knowledge assessment per section. For each of the sections covered during the training, we observed a statistically significant mean difference in the pre and post-test knowledge assessment for the research questions, objectives, and aim (p=0.003), introduction to clinical trials (p=0.014) and stepped wedge study design (p=0.037). Table 3 describes knowledge assessment per question. We observed statistical significance for four questions: Q1, Q6, Q9 and Q17 (all p < 0.044).

**Table 2:** knowledge assessment of participants per section covered during the training

Section	Pre-test	Post-test	Mean difference	p-value
	Mean score (SD)	Mean score (SD)		
Research questions, objectives, and aim	2.0 (0.9)	2.6 (0.5)	0.6	**0.003**
Research study design	1.8 (1.1)	1.9 (1.1)	0.1	0.78
Introduction to clinical trial	2.4 (0.6)	2.8 (0.5)	0.3	**0.01**
Stepped wedge study design	1.8 (0.9)	2.2 (0.6)	0.4	**0.04**
Descriptive statistics	0.5(0.5)	0.6 (0.5)	0.1	0.65
Scientific writing	4.4(0.8)	4.7 (0.5)	0.2	0.07

**Table 3:** knowledge assessment of participants per question covered during the training

Question No	Pre-test		Post-test		Mean difference	% Change*	p-value
	N (%) n=38	Mean score (SD)	N (%) n=33	Mean score (SD)			
1	20 (52.6)	0.53 (0.51)	31 (93.9)	0.94 (0.24)	0.41	Good to Excellent	<0.001
2	22 (57.9)	0.58 (0.50)	23 (69.7)	0.69 (0.47)	0.11	Good to Good	0.26
3	38 (100)	1 (0)	33 (100)	1 (0)	0	Excellent to Excellent	0.74
4	22 (57.9)	0.58 (0.50)	19 (57.6)	0.58 (0.50)	0	Good to Good	0.79
5	26 (68.4)	0.68 (0.47)	21 (63.6)	0.64 (0.49)	-0.04	Good to Good	0.54
6	24 (63.2)	0.63 (0.49)	22 (66.7)	0.67 (0.48)	0.04	Good to Good	0.002
7	20 (52.6)	0.54 (0.51)	29 (87.9)	0.88 (0.33)	0.34	Good to Excellent	0.57
8	37 (97.4)	0.97 (0.16)	31 (93.9)	0.94 (0.24)	-0.03	Excellent to Excellent	0.25
9	38 (100)	1 (0)	33 (100)	1 (0)	0	Excellent to Excellent	0.04
10	23 (60.5)	0.61 (0.49)	24 (72.7)	0.73 (0.45)	0.12	Good to Good	0.14
11	34 (89.5)	0.89 (0.31)	33 (100)	1 (0)	0.11	Excellent to Excellent	0.65
12	12 (31.6)	0.32 (0.47)	15 (45.5)	0.45 (0.51)	0.13	Average to Average	0.16
13	20 (52.6)	0.53 (0.51)	19 (57.6)	0.58 (0.50)	0.05	Good to Good	0.08
14	36 (94.7)	0.95 (0.226)	33 (100)	1 (0)	0.05	Excellent to Excellent	1.00
15	33 (86.8)	0.87 (0.343)	33 (100)	1 (0)	0.13	Excellent to Excellent	0.33
16	35 (92.1)	0.92 (0.273)	31 (93.9)	0.94 (0.24)	0.02	Excellent to Excellent	0.49
17	36 (94.7)	0.95 (0.226)	33 (100)	1 (0)	0.05	Excellent to Excellent	<0.001
18	24 (63.2)	0.63 (0.489)	24 (72.7)	0.73 (0.45)	0.10	Good to Good	0.26

* 0-25% = Zero, 26-50% = Average, 51-75% = Good, 76-100% = Excellent

### Evaluation of self-efficacy

**Figure 1:**
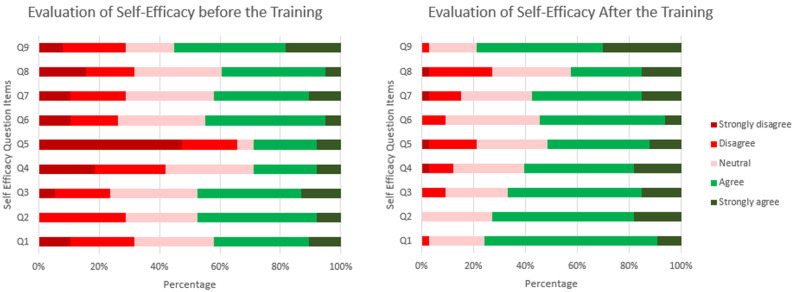
evaluation of self-efficacy before and after training

Overall, for the nine items (Q1-Q9) on self-efficacy, the mean (SD) pre-test and post-test scores were 2.9 (0.9) and 3.7 (0.7), respectively. We observed a statistically significant mean score difference of 0.8 (p < 0.001) for the participants´ self-confidence of their knowledge improvement after the training. Table 4 describes the evaluation of self-efficacy per item. The belief of knowledge improvement was statistically significant for almost all the self-efficacy items (except Q8). There was a greater difference in the perceived change in knowledge upon training for stepped-wedge study design (Q5) with a mean difference of 1.2 (p < 0.001). The stacked bar diagram in Figure 1 illustrates the overall shift in the perception participants had of their knowledge concerning research methodology topics. The colour shift describes the transition from complete disagreement (dark red) to complete agreement (dark green) in self-efficacy before and after training for each item statement. Every item showed a positive trend shift from strong disagreement (dark red) and disagreement (red) to agreement (green) and strong agreement (dark green). The most noticeable change from disagreement (dark red and red) to agreement (dark green and green) was observed for Q1 (formulating research questions), Q2 (clearly write research questions, objectives and aims), Q5 (stepped wedge study design) and Q9 (scientific paper writing).

**Table 4:** evaluation of self-efficacy per item

Item	Pre-test		Post-test		Mean difference	P-value
	Mean	Standard deviation	Mean	Standard deviation		
1	3.11	1.18	3.82	0.64	0.7	**0.002**
2	3.26	0.98	3.91	0.68	0.7	**0.002**
3	3.32	1.09	3.73	0.84	0.4	**0.04**
4	2.76	1.22	3.64	0.99	0.9	**0.001**
5	2.24	1.44	3.39	1.03	1.2	**<0.001**
6	3.13	1.09	3.52	0.76	0.4	**0.02**
7	3.13	1.17	3.55	1.00	0.4	**0.04**
8	2.97	1.17	3.27	1.10	0.3	0.09
9	3.37	1.2	4.06	0.79	0.7	**0.003**

## Discussion

All participants completed the pre-test, while two participants had to leave earlier and did not complete the post-test. Overall, our findings suggest that the training had a positive effect on both the knowledge and self-efficacy of participants regarding research methodology. Importantly, these results highlighted serious gaps in knowledge of participants on research methods but also confirmed the huge potential of improvement through simple actions. Our study serves as a proof-of-concept, demonstrating the feasibility and potential impact of such training programs. The identified knowledge gaps underscore the need for concerted efforts by governments and international organizations to enhance health science research capacity. Our findings contribute to the broader discourse on improving health science research, particularly in contexts lagging behind other countries or regions.

We noted that more than half of participants in this study were women (56.7%) and the median (IQR) age of 33.5 (13.3) years. Most participants reported to have completed a master´s level. This suggests that most participants in our study were not long-experienced field researchers, but already had good baseline knowledge on research methodology. Similarly, a majority reported to have already taken part in a research training before and had already been involved in writing of abstracts or manuscripts. This could suggest the familiarity of participants with research methods theoretical and practical concepts. This equally explains the relatively high mean score value on knowledge assessment for the pre-test. Compared to other similar studies whose participants were mostly health students (MD, pharmacists, nurses), graduates and post graduates [6-11], our study included all health professionals, researchers as well as graduates. Our study showed a marked overall improvement in the participants' knowledge observed with a mean score improvement of 1.8 (p=0.001). Similarly, some previous studies reported an increase in knowledge assessment after workshops with a 30% increase [12] and 20.6% increase [13]. Participants' knowledge increased significantly for the topics on research questions objectives and aims, introduction to clinical trial, and stepped wedge study design as compared to the topics on research study design, descriptive statistics and scientific writing which did not show a significant difference. This suggests that we need more time to train and to incorporate more practical exercises and case studies for these components.

The overall mean score of the participants´ confidence in their efficacy on research methods increased from 2.9 to 3.7 (p < 0.001) from the pre to the post-test. This is evidence on the impact that the training has on the confidence of individuals regarding their skills and capacities to produce research studies of reliable and good quality. It also suggests that participants believed the training added some value to their knowledge as concerns research methodology. Self-efficacy was also reported to have increased after workshops for other studies [6-8,14]. A study on clinical research training with MD/PhD students showed that participants exposed to a training had a higher self-efficacy than those who were not [9]. Finally, the most appreciable changes in self-efficacy (from deep red to dark green), as observed in Figure 1, on stepped-wedge study design suggests that this topic was new to most participants and the training stepped up their knowledge on this study design which is of increasing use in research [15]. Also, we observe a marked shift from deep red to dark green for the item on scientific writing (Q9) which is similar to a finding in a nationwide virtual research workshop in Pakistan [13].

Some limitations of this study were as follow; Firstly, the duration of the training was not long enough to cover the broad range of topics in research methods, and consequently our participants were evaluated only in areas they received the training on. Therefore, there is a risk that the proxies we used do not capture the full landscape of the needs in research methods that we sought to map the possibility of improvement. Secondly, the small sample size prevented us to study factors that influenced the level of knowledge as well as the potential of improvement after a health research method training. Lastly, the improvement observed in this small, selected sample of participants was an immediate change and we cannot guarantee the sustainability of the effect. A longer follow-up is necessary to assess the impact of the training on research capacities of our participants.

## Conclusion

The results obtained from our study suggests that knowledge of the participants who attended the CAM-HERO 2022 training on research methodology was significantly improved and there was marked change in the perception of their self-efficacy on research aptitudes across the different topics taught in the training. Hence, implementing, and adapting research methodology trainings can play an important role in acquiring and refining knowledge and skills among researchers. Governments and international organizations must encourage and fund such short course trainings on a larger scale to strengthen health sciences research capacity in African countries including Cameroon.

## Annex

**Annex 1**: program of the research methodology training (PDF-431 KB)
